# Pre-mRNA processing factors meet the DNA damage response

**DOI:** 10.3389/fgene.2013.00102

**Published:** 2013-06-06

**Authors:** Alessandra Montecucco, Giuseppe Biamonti

**Affiliations:** Istituto di Genetica Molecolare, Consiglio Nazionale delle RicerchePavia, Italy

**Keywords:** pre-mRNA processing, RNA binding proteins, splicing, DNA damage response, checkpoint kinases

## Abstract

It is well-known that DNA-damaging agents induce genome instability, but only recently have we begun to appreciate that chromosomes are fragile *per se* and frequently subject to DNA breakage. DNA replication further magnifies such fragility, because it leads to accumulation of single-stranded DNA. Recent findings suggest that chromosome fragility is similarly increased during transcription. Transcripts produced by RNA polymerase II (RNAPII) are subject to multiple processing steps, including maturation of 5′ and 3′ ends and splicing, followed by transport to the cytoplasm. RNA maturation starts on nascent transcripts and is mediated by a number of diverse proteins and ribonucleoprotein particles some of which are recruited cotranscriptionally through interactions with the carboxy-terminal domain of RNAPII. This coupling is thought to maximize efficiency of pre-mRNA maturation and directly impacts the choice of alternative splice sites. Mounting evidence suggests that lack of coordination among different RNA maturation steps, by perturbing the interaction of nascent transcripts with the DNA template, has deleterious effects on genome stability. Thus, in the absence of proper surveillance mechanisms, transcription could be a major source of DNA damage in cancer. Recent high-throughput screenings in human cells and budding yeast have identified several factors implicated in RNA metabolism that are targets of DNA damage checkpoint kinases: ATM (ataxia telangiectasia mutated) and ATR (ATM-Rad3 related) (Tel1 and Mec1 in budding yeast, respectively). Moreover, inactivation of various RNA processing factors induces accumulation of γH2AX foci, an early sign of DNA damage. Thus, a complex network is emerging that links DNA repair and RNA metabolism. In this review we provide a comprehensive overview of the role played by pre-mRNA processing factors in the cell response to DNA damage and in the maintenance of genome stability.

Mounting evidence collected over the last few years supports the idea that RNA-binding proteins (RBPs) involved in different steps of mRNA life, from transcription to translation, can affect genome stability programs (Matsuoka et al., 2007; Paulsen et al., 2009; Hurov et al., 2010). In particular, a number of large-scale genetic and proteomic quests for proteins involved in the DNA damage response (DDR) have revealed enrichment in RNA processing proteins, indicating that RNA metabolism and DNA repair pathways functionally intersect. However, the role played by mRNA processing factors in the cell response to endogenous and exogenous sources of DNA damage is still largely unexplored.

In this review, after a short introduction on the basic principles of pre-mRNA splicing, we will discuss genome-wide approaches implicating RBPs in the DDR. Thereafter, we focus on three particular aspects:

1) RNA-binding proteins may affect the splicing profiles and levels of mRNAs for proteins involved in the cell response to DNA damage. In this section, we address important facets such as (i) the role of splicing in apoptosis; (ii) the redistribution of splicing factors as a strategy to control splicing programs after DNA damage; (iii) the modulation of mRNA stability; (iv) the importance of cotranscriptional splicing, and (v) post-translational modifications of RBPs in the DDR.
2) RNA-binding proteins may directly participate in the DDR. A few examples will be provided to illustrate this still poorly understood phenomenon.
3) RNA-binding proteins may prevent DNA damage. Once pre-mRNA has been transcribed, it is processed into mature ribonucleoprotein (mRNP) particles. In this section, we discuss the role of mRNP biogenesis factors in preventing hazardous R-loops.

Finally, we speculate on the potential role that RBPs may play in the effect of programmed DNA damage on cell differentiation, a poorly understood subject.

## PRE-mRNA PROCESSING

**FIGURE 1:**
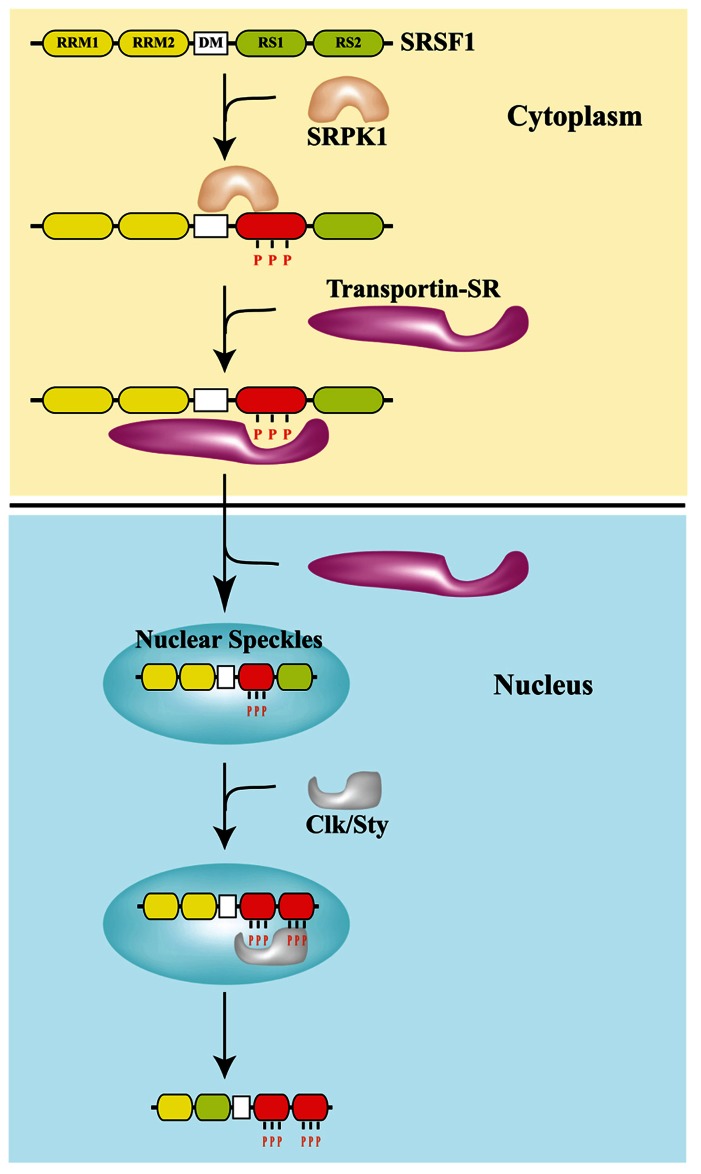
**Phosphorylation controls the subcellular distribution of splicing factor SRSF1.** The arginine–serine (RS)-rich domain of the SR protein SRSF1 is phosphorylated by SR protein kinases SRPK and Clk/Sty. The docking motif (DM) restricts phosphorylation of SRSF1 by SRPK1 at the N-terminal portion of the RS domain (RS1), which is required for the interaction with transportin SR and nuclear import. In the nucleus, SRSF1 accumulates in nuclear speckles. Clk/Sty causes release of SRSF1 from speckles by phosphorylating the C-terminal portion of its RS domain (RS2; Ngo et al., 2005).

The majority of metazoan genes consist of an ordered succession of coding (exon) and non-coding (intron) sequences. The generation of translatable mRNAs requires the precise removal of intronic sequences *via* a complex multistep reaction known as splicing. This reaction is carried out by the spliceosome, a large molecular machine, composed of five small nuclear ribonucleoproteins (snRNPs U1, U2, U4, U5, and U6) and more than 100 different polypeptides (Wahl et al., 2009). The spliceosome recognizes short, poorly conserved, *cis*-acting sequence elements at exon–intron boundaries (5′ and 3′ splice sites) and uses these to remove the intron through two sequential trans-esterifications. Alternative splicing events, using various combinations of donor and acceptor sites from different exons, produce more than one mRNA molecule from a single pre-mRNA. Five distinct alternative splicing patterns have been observed: (1) cassette exons, which may be either selected or skipped during the generation of mRNA; (2) mutually exclusive exons; (3) intron retained; (4) alternative donor, and (5) acceptor sites which alter the length of exons. Moreover, alternative promoters and poly-adenylation sites contribute to the heterogeneity of transcripts encoded by a single gene (Ghigna et al., 2008). In addition to modifying protein features, alternative splicing can also affect the stability of transcripts by introducing premature STOP codons, thus directing mRNA degradation through the non-sense-mediated mRNA decay (NMD) pathway (Maquat and Carmichael, 2001). This regulatory mechanism frequently operates to control the homeostatic level of genes encoding most RBPs, particularly splicing regulators (Valacca et al., 2010). Alternatively spliced exons are usually flanked by short and degenerate splice sites, and their recognition is modulated by regulatory sequences referred to as enhancers and silencers of splicing that respectively promote and inhibit exon recognition. These elements are present both within exons (ESEs, exonic splicing enhancers and ESSs, exonic splicing silencers) and introns (ISEs, intronic splicing enhancers and ISSs, intronic splicing silencers; Black, 2003). Enhancers function by providing binding sites for serine–arginine (SR) factors, a family (about a dozen) of essential and abundant RBPs highly conserved in evolution (Cartegni et al., 2002). SR factors display multiple roles in constitutive and alternative splicing, as well as in other aspects of gene expression (Hastings and Krainer, 2001). They share a modular structure consisting of one or two copies of an RNA-recognition motif (RRM) at the N-terminus followed by a carboxy-terminal domain of variable length rich in alternating SR dipeptides (the RS domain). The RRMs determine RNA-binding specificity, whereas the RS domain mainly mediates specific protein–protein interactions that are essential for the recruitment of the splicing apparatus. In addition, RS domains are targets of phosphorylation events that influence protein interactions (Xiao and Manley, 1998), and regulate the activity and subcellular distribution of SR proteins (Gui et al., 1994; see **Figure 1**). Several kinases, including SR protein kinases (SRPKs) 1 and 2, Clk/Sty, dual-specificity tyrosine-regulated kinase, DNA topoisomerase I (Topo I), glycogen synthase kinase-3 and AKT/Protein Kinase B, have been shown to phosphorylate SR proteins (for a review see Ghigna et al., 2008). However, the signal transduction pathways that regulate the activity of these kinases and their role in alternative splicing are still poorly understood.

Splicing silencers may act as binding sites for factors that block splicing machinery access to a splice site. Proteins that interact with silencer elements include heterogeneous nuclear ribonucleoproteins (hnRNP), a group of RBPs that interact with RNA polymerase II (RNAPII) transcripts to form hnRNP particles (Black, 2003). Similar to SR factors, hnRNP proteins have a modular structure in which one or more RNA binding domains, generally at the N-terminus, are associated with different “auxiliary” domains. Three types of RNA binding domains (RRMs, hnRNP K homology – KH – domain and RGG domain, a protein region rich in Arg-Gly-Gly) have been described; these provide a certain level of RNA binding specificity (Black, 2003). The auxiliary domains are very different in sequence and control the subcellular localization and interaction with other proteins. Altogether, RNA binding specificity and protein–protein interactions contribute to the cotranscriptional assembly of hnRNP complexes that are the substrates of the splicing reaction. In addition to SR factors and hnRNP proteins, a number of tissue-specific splicing regulators have been identified some of which, i.e., RNA binding protein fox-1 homolog (C.elegans) 1 and 2 (RBFOX1 and 2) and neuro-oncological ventral antigen (NOVA), bind to specific RNA sequence elements (Black, 2003).

The vast majority of alternative splicing events are controlled by the relative abundance and/or activity of widely expressed antagonistic SR factors and hnRNP proteins through a combinatorial mechanism, with multiple positive and negative factors and sequence elements influencing the final outcome of the splicing reaction. A classical example is the antagonistic activity of SRSF1 (serine/arginine-rich splicing factor 1), an SR factor, and hnRNP A1: high levels of SRSF1 induce exon inclusion, whereas high levels of hnRNP A1 promote exon skipping (Cáceres et al., 1994). Recent studies indicate that signaling pathways may control splicing decisions by affecting the subcellular distribution and/or activity of splicing regulators (Shultz et al., 2010). Many SR factors and hnRNP proteins continuously and rapidly shuttle between the nucleus and the cytoplasm (Cáceres et al., 1998), which reflects their involvement in several aspects of RNA life from transcription to translation.

Alternative splicing is a highly pervasive mechanism of gene expression regulation that affects the vast majority (more than 90%) of human genes (Pan et al., 2008). It is not surprising, therefore, that also transcripts encoding factors involved in the DDR, checkpoint or apoptosis undergo alternative splicing events that affect protein function in response to conditions of stress. However, very few genome-wide analyses on the effects of DNA damage on splicing profiles have been performed to date and we still have a very fragmented view of the logic underling this regulatory mechanism. For instance, only recently the first comprehensive characterization of human transcriptome changes occurring in response to ionizing radiation (IR) in human lymphoblastoid cell lines was reported (Sprung et al., 2011).

## LARGE-SCALE GENETIC AND PROTEOMIC ANALYSES

Several unbiased large-scale genetic and proteomic screenings in the last few years have revealed a connection between pre-mRNA processing and genome stability programs. For instance, a proteomic analysis designed to identify human and mouse proteins phosphorylated in response to DNA damage on ATM (ataxia telangiectasia mutated) and ATR (ATM-Rad3 related) consensus sites, revealed about 700 targets. Most of these belong to pathways not previously implicated in the response to DNA damage, and include factors with a role in RNA metabolism. The list of validated targets includes RBM10 (RNA binding motif protein 10), which associates with hnRNP complexes and is required for phosphorylation of the histone variant H2AX after IR (Matsuoka et al., 2007). A similar screening in yeast (Smolka et al., 2007) led to the identification of nuclear protein localization 3 (Npl3), a protein related to human SRSF1 and hnRNP A1, which is also involved in mRNA export to the cytoplasm. In the same assay, another splicing factor, called PRP19, was identified which has a direct role both in the DDR and in preventing DNA damage induced by transcription.

Another proteomic analysis quantified DNA damage-regulated changes in phosphoproteome, acetylome, and proteome in human osteosarcoma cells treated with etoposide, a topoisomerase II (Topo II) inhibitor that causes double-stranded DNA breaks (DSBs; Beli et al., 2012). Also in this case a significant fraction of the hits corresponded to proteins involved in RNA metabolism. The same authors focused on the RNA processing factor THRAP3 (thyroid hormone receptor-associated protein 3), which is part of a multiprotein complex that controls Cyclin D1 mRNA stability, and the splicing-regulator phosphatase protein phosphatase Mg2+/Mn2+ dependent 1G (PPM1G), a nuclear member of the PP2C family of Ser/Thr phosphatases. Phosphorylation of THRAP3 mainly depends on the activity of ATR and is elicited by various DNA-damaging agents and by the DNA replication inhibitor hydroxyurea. Importantly, THRAP3 down-regulation makes cells more sensitive to fork stalling, but the molecular mechanism underlying this effect is still undefined. The lack of colocalization with γH2AX foci suggests that THRAP3 may play an indirect role in the DDR, for instance, by regulating alternative splicing or mRNA stability of transcripts for proteins involved in DDR, checkpoints or cell cycle progression.

The notion that RNA processing and DNA repair functionally intersect has been recently bolstered by a genome-wide small interfering RNA (siRNA)-based screening for regulators of homologous recombination (HR; Adamson et al., 2012). This study identified a number of pre-mRNA processing proteins among positive regulators of HR, while phosphatase networks were included in the list of negative regulators.

**FIGURE 2:**
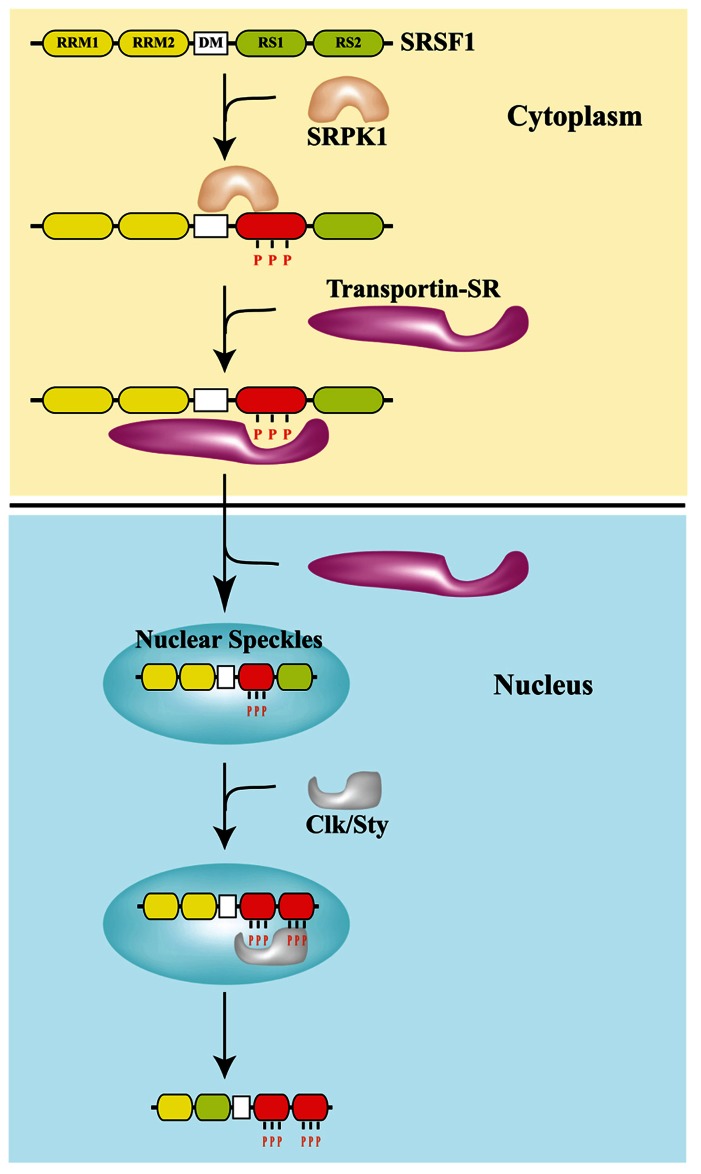
**DNA damage affects splicing decisions by modulating the phosphorylation status of RNAPII and the elongation rate of transcription.** CPT-induced Top1ccs have immediate and specific effects on RNAPII. CPT triggers a high degree of phosphorylation of the largest subunit (Rpb1) of RNAPII (Baranello et al., 2010). Ultraviolet (UV) irradiation affects cotranscriptional alternative splicing in a p53-independent manner, through hyperphosphorylation of the RNAPII carboxy-terminal domain (CTD) and subsequent inhibition of transcriptional elongation (Muñoz et al., 2009).

Finally, a genome-wide approach was applied by Yves Pommier and colleagues to study the effect of the Topo I inhibitor camptothecin (CPT) on splicing decisions in human colon carcinoma HCT116 and breast carcinoma MCF7 cells. CPT preferentially affects splicing of transcripts for splicing factors, such as RBM8A, which belongs to the protein complex that tags exon–exon junctions after the splicing reaction (Solier et al., 2010). Interestingly, they showed that the production of the Topo I–DNA cleavage complex – Top1cc – triggered by CPT slows down RNA elongation through the rapid hyperphosphorylation of RNAPII and affects splicing profiles. The effect on the elongation rate of RNAPII and splicing programs appears to be an outcome shared with other DNA-damaging agents, such as ultraviolet (UV) irradiation (Muñoz et al., 2009). Two alternative models have been proposed to explain the link between the RNAPII elongation rate and splicing programs. In the “kinetic coupling model” a slow RNAPII may favor the usage of weak splice sites (de la Mata et al., 2010; **Figure 2**). Alternatively, hyperphosphorylation of the CTD of RNAPII may affect the recruitment of splicing factors to the transcriptional machinery as proposed in the “recruitment coupling model” (Listerman et al., 2006).

## RBPs MAY AFFECT THE SPLICING PROFILES AND LEVELS OF mRNAs FOR FACTORS INVOLVED IN THE CELL RESPONSE TO DNA DAMAGE

### SPLICING AND APOPTOSIS

A large amount of evidence implicates splicing decisions in the choice between cell survival and apoptosis in response to DNA damage. The functional consequence of alternative splicing on apoptosis has been documented for many genes, including cell surface receptors, such as Fas; adaptor proteins and regulators, including TRAF2 (TNF receptor-associated factor 2) and APAF-1 (apoptotic protease activating factor 1); mediators, such as B-cell lymphoma-extra (Bcl-x), Bcl-2 homologous antagonistic/killer (Bak), and myeloid cell leukemia sequence 1 (Mcl-1); and caspases. Remarkably, mRNAs encoding some members of the Bcl2 family of apoptotic factors, Bcl-x and Mcl-1, are alternatively spliced to yield both large (L) anti-apoptotic and short (S) pro-apoptotic forms. The choice between these alternatives has been investigated in several studies that reported the identification of relevant sequence elements and RBPs (reviewed in Moore et al., 2010).

Among the known inducers of apoptosis, Ceramide was the first one shown to control splicing of transcripts encoding members of the Bcl-2 (B-cell CLL/lymphoma 2) family and caspase 9. Ceramide treatment increases the level of pro-apoptotic splice variants Bcl-x(S) and caspase 9a, with a concomitant loss in the anti-apoptotic Bcl-x(L) and caspase 9b isoforms. This effect involves the regulation of the phosphorylation status of SR splicing factors, including SRSF1 (Massiello and Chalfant, 2006), through activation of PP1 phosphatase (Chalfant et al., 2002). The importance of SRSF1 phosphorylation in splicing of caspase 9 transcripts is indicated also by the observation that post-translational modification of this factor by the signaling kinase AKT promotes caspase 9b production (Shultz et al., 2010).

Insights into the molecular mechanisms that control these splicing events came from the analysis of the response to the genotoxic stress induced by oxaliplatin. This compound elicits an ATM-, CHK2 (checkpoint kinase 2)-, and p53-dependent splicing switch that favors the production of the pro-apoptotic Bcl-x(S) variant and acts through a regulatory sequence element called SB1. Surprisingly, the same SB1 element mediates the accumulation of the larger anti-apoptotic Bcl-x(L) isoform upon activation of the PKC pathway. Thus, one splicing regulatory module can receive antagonistic signals from the PKC and the p53-dependent DNA damage response pathways to control the balance of pro- and anti-apoptotic Bcl-x splice variants (Shkreta et al., 2011) underlining the complexity of the regulatory circuits that orchestrate the cell response to conditions of stress.

In a landmark paper, Pamela Silver applied a genome scale siRNA screening to search for new regulators of Bcl-2 pre-mRNA splicing. The list of regulators identified by this screening appears to be enriched not only for splicing but also for cell cycle functions. Interestingly, treatments that induce mitotic arrest by targeting the mitotic aurora kinase A(AURKA) kinase promote the coordinated pro-apoptotic splicing of Bcl-x, Mcl1, and caspase-9 suggesting the existence of an alternative splicing network that links cell cycle control to apoptosis. Upon AURKA knockdown or inhibition, only splicing factor SRSF1 was down-regulated, most likely via modulation of post-translational turnover (Moore et al., 2010). Notably, the SRSF1 function as an inhibitor of apoptotic pathways and promoter of cell survival is in line with the oncogenic potential of this splicing regulator (Karni et al., 2007). Collectively these analyses indicate that perturbations of the post-translational modification profile and expression level of SRSF1 may impact Bcl-2 pre-mRNA resulting in the production of pro-apoptotic isoforms (Moore et al., 2010).

### REDISTRIBUTION OF SPLICING FACTORS

Although SR splicing factors and hnRNP proteins are commonly considered nuclear proteins most of them continuously shuttle between the nucleus and the cytoplasm, a property that reflects their role both in mRNA export and in translation (Weighardt et al., 1995; Cáceres et al., 1998). Nuclear re-import requires the interaction with dedicated import proteins and in the case of SR factors depends on the phosphorylation of specific residues in the RS domain by SRPK1 and 2 kinases (**Figure 1**). In addition to being distributed throughout the nucleoplasm, SR factors display a characteristic accumulation in highly dynamic nuclear sub-compartments known as splicing speckles that are viewed as depots for proteins involved in splicing. Phosphorylation by Clk/Sty mobilizes SR factors from nuclear speckles to the nucleoplasm where transcription and mRNA maturation occurs in the perichromatin compartment (Biamonti and Caceres, 2009). One of the strategies exploited by the cells to modulate splicing decisions in response to stress conditions, including DNA damage, is the redistribution of splicing factors. We significantly contributed to the identification of this strategy by showing that heat shock, heavy metals and osmotic stress, which threaten genome integrity, influence the sub-nuclear distribution of specific splicing factors. We have shown that splicing factors SRSF1, SRSF9, hnRNP K, Saf-B (scaffold attachment factor B), and Sam68 (Src-associated substrate in mitosis of 68 kDa) are recruited to transcription sites of repetitive genomic DNA in areas called nuclear stress bodies (Biamonti and Vourc’h, 2010). Osmotic stress also produces the accumulation of a subset of hnRNP proteins, including hnRNP A1, into cytoplasmic stress granules. This involves the phosphorylation of hnRNP A1 by the Mnk1/2 protein kinases that act in the p38 stress-signaling pathway (Guil et al., 2006; Biamonti and Caceres, 2009). Another example of the stress effect on cellular distribution of splicing factors is hSlu7, which plays an important role in 3′ splice site selection during the second step of splicing *in vitro*. It has been shown that UV irradiation decreases the nuclear concentration of hSlu7 through the modulation of its nucleus-to-cytoplasm transport. This shift is mostly dependent on the Jun N-terminal kinase cascade. Moreover, the nuclear concentration of hSlu7 affects exon choice and alternative splicing programs (Shomron et al., 2005). More recently it was shown that mitoxantrone, a Topo II inhibitor, induces relocalization of several RBPs, such as TIA-1, hnRNP A1, SRSF1, and SRSF2, from the nucleoplasm to nuclear granules that serve as transcriptional factories, even though the identity of the transcribed genes has not yet been defined. This redistribution is independent of signal transduction pathways activated by DNA damage and is accompanied by changes in the alternative splicing programs of target genes such as antigen (CD44; Busà et al., 2010). Numerous other RBPs have been reported to change their distribution in response to a variety of stress conditions; however, the mechanisms involved in these redistributions have not been investigated. Thus, we still have a very superficial and fragmented description of this regulatory strategy that could be part of the cell response to DNA damage.

### MODULATION OF mRNA STABILITY PROGRAMS

DNA damage elicits the activation of signaling networks, identified by apical kinases ATM and ATR, leading to the rapid phosphorylation of a large set of cellular proteins. The ultimate function is to produce an immediate arrest of the cell cycle along with recruitment of the repair machinery to damaged DNA. The list of targets for these signaling pathways also includes transcription factors, in particular p53, whose activation drives a delayed transcriptional response aimed at promoting cell cycle arrest through the induction of Cdk (cyclin-dependent kinase) inhibitors, i.e., p21, and which presides over the choice between cell survival and apoptotic pathways. A number of studies have recently identified a third intermediate branch of the DDR that operates on post-transcriptional regulatory circuits such as alternative splicing and mRNA stability programs. In this branch, RBPs would serve both as targets of the signaling network elicited by DNA damage and as transducers of signals to downstream gene expression programs.

A significant fraction of mRNAs is either up- or down-regulated after cell exposure to IR, UV, or treatment with MMS (methylmethane sulphonate; Rieger and Chu, 2004). These changes involve manipulation of mRNA stability through modulation of the interactions between RBPs and their target mRNA molecules.

One example is the mRNA encoding the growth arrest- and DNA damage-inducible GADD45α protein, which is potently up-regulated in response to stress stimuli. Two RBPs are critical negative regulators of GADD45α mRNA and protein levels: AUF1, which targets GADD45α mRNA for degradation, and T-cell-restricted intracellular antigen (TIA) 1-related protein (TIAR), which prevents the association of GADD45α mRNA with translating polysomes. The interaction of these two proteins with the 3′-untranslated region (UTR) of the GADD45α mRNA in HeLa cells drastically decreases after exposure to UV or treatment with MMS. Crucial for this response is the signaling pathway identified by p38 and MAPKAP kinase-2 (p38/MK2) that operates in the cytoplasm downstream of ATM and ATR. p38/MK2 modulates mRNA stability through phosphorylation of RNA-binding/regulatory proteins, including hnRNPA0, TIAR, and polyA-specific ribonuclease (PARN), and leads to stabilization of mRNAs containing AU-rich elements in their 3′-UTR (Reinhardt et al., 2010).

In addition to GADD45α mRNA, numerous transcripts are substrates of this regulatory mechanism, a significant fraction of which encodes for proteins relevant to cell cycle control. For example HuR and hnRNP C1 bind diverse AU-rich elements in the 3′-UTR of the p21 transcript and function cooperatively to stabilize p21 mRNA in response to UV, gamma radiation, and other stress causing treatments (Cho et al., 2010). In contrast, the PCBP (poly(C)-binding protein) family of RBPs, composed of five major members hnRNP K, PCBP1, PCBP2, PCBP3, and PCBP4, binds CU-rich elements in the 3′-UTR to negatively regulate p21 expression (Waggoner et al., 2009; Scoumanne et al., 2011).

### COTRANSCRIPTIONAL PROCESSING AND SPLICING

Many pre-mRNA processing factors are recruited to the RNA molecule cotranscriptionally through poorly characterized protein interactions that involve the CTD of RNAPII (Das et al., 2006). As a consequence, nascent pre-mRNA molecules emerging from the transcriptional apparatus are immediately assembled into ribonucleoprotein (RNP) complexes that constitute the substrate of the splicing reaction and determine splicing decisions. The protein moiety of these complexes depends on several parameters such as the sequence specificity of binding of splicing factors (in most cases relatively poor) and protein–protein interactions established among RBPs that are fine-tuned by post-translational modifications. Moreover, the modulation of the RBP interactions with RNAPII and the elongation rate of transcription, have been shown to play a role in splicing decisions. Although the splicing reaction does not necessarily occur cotranscriptionally, the cotranscriptional recruitment of RBPs may enhance the efficiency of the process.

In a seminal paper published a few years ago, Kornblihtt showed that UV affects alternative splicing profiles in a p53-independent way. This effect requires the hyperphosphorylation of the CTD of RNAPII, which leads to inhibition of transcriptional elongation, a condition known to favor inclusion of alternative exons by allowing enough time for the usage of weak splice sites (see **Figure 2**). Consistently, gene expression analyses with a splicing sensitive array evidenced a significant overlap between gene transcripts undergoing changes in alternative splicing after UV, and those with reduced expression (Muñoz et al., 2009).

**FIGURE 3:**
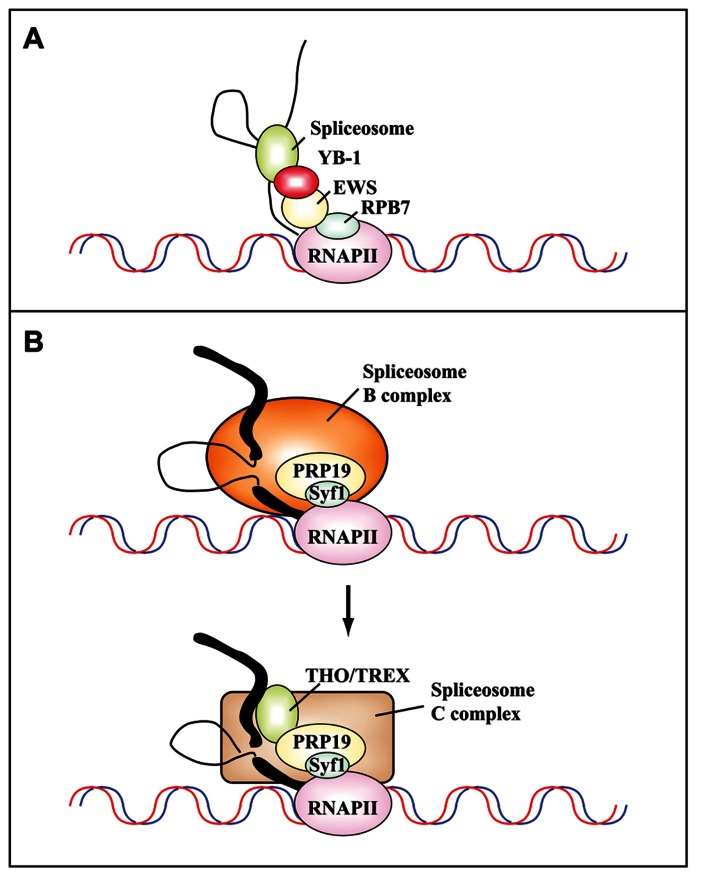
**Protein–protein interactions mediate the cotranscriptional assembly of ribonucleoprotein complexes that are targets of DNA damage-induced signaling pathways.** **(A)** Schematic representation of the communication between the transcriptional and splicing machineries mediated by RPB7, EWS, and YB-1. Camptothecin inhibits the interaction between Ewing’s sarcoma proto-oncoprotein (EWS), an RNAPII-associated factor, and YB-1, a spliceosome-associated factor. This results in the cotranscriptional skipping of several exons of the *MDM2 *gene (Dutertre et al., 2010). **(B)** The PRP19 complex functions in transcription and is recruited to the transcription machinery by the C terminus of its component Syf1, the yeast homolog of human XAB2 (adapted from Chanarat et al., 2011). Human XAB2 co-purifies both with factors involved in transcription (RNAPII), splicing (PRP19), and TCR (XPA, CSA and CSB; Kuraoka et al., 2008). The PRP19 complex is required for the recruitment of the THO/TREX complex to nascent transcripts after the switch from the B to the C splicing complex. Thick and thin black lines represent exons and introns, respectively.

A completely different mechanism of cotranscriptional regulation of splicing profiles has been shown to operate in response to cell treatment with the Topo I inhibitor CPT (Dutertre et al., 2010). Among the 354 exons that are skipped after a short CPT treatment, Auboeuf and colleagues focused on the splicing program of transcripts for MDM2, an E3 ubiquitin ligase that targets p53 for proteasomal degradation. Notably, in addition to CPT, a number of well-known genotoxic stressors, including doxorubicin and cisplatin, can promote* MDM2 *exon skipping (Dutertre et al., 2010). CPT acts by disrupting the interaction between EWS (Ewing’s sarcoma proto-oncoprotein), an RNAPII-associated factor, and YB-1 (Y box binding protein 1), a spliceosome-associated factor (see **Figure 3A**). This is the first demonstration that stress treatment can alter the communication between transcription and splicing machineries leading to exon skipping and provides a good molecular model for the rapid regulation of splicing programs in response to stress, as shown in yeast (Pleiss et al., 2007).

The EWS protein is a member of the TET family (TLS/FUS, EWS, and TAF15) of RBPs and DNA-binding proteins, and functions both in transcription and RNA processing. It is involved in HR, the DDR, and maintenance of genome integrity and its knock-out induces a phenotype similar to that observed upon knock-out of ATM. Furthermore, the EWSR1 (Ewing sarcoma breakpoint region 1) gene product is important for resistance to IR (Hurov et al., 2010). Thus, EWS acts as a bridge between transcription and splicing machineries: it interacts with proteins involved in transcription, such as the pre-initiation complex TFIID and subunits of the RNAPII, and with splicing factors, including the U1 snRNP protein U1C, the branch-point binding protein BBP/SF1, and the spliceosome component YB-1 (see references in Dutertre et al., 2010; Paronetto et al., 2011). UV-light induces dissociation of EWS from sites of active transcription, and therefore affects the alternative splicing of genes regulated by this protein such as ABL1, CHK2, and MAP4K2 that are important for the response to cell stress and DNA damage (Paronetto et al., 2011). This is accompanied by the transient enrichment of EWS in nucleoli, which provides a further example of redistribution of factors as an efficient strategy used by the cells to reprogram gene expression after treatment with DNA-damaging agents.

### POST-TRANSLATIONAL MODIFICATION OF RBPs AND DDR

Recently, we have investigated the cell response to chronic replication-dependent DNA damage in human DNA ligase I (LigI)-deficient cells. The LigI defect hampers the maturation of Okazaki fragments and results in the accumulation of single-stranded DNA breaks (SSBs) and DSBs and in the constitutive activation of the ATM checkpoint pathway (Soza et al., 2009). By applying a proteomic approach, we have shown that the entire set of SR splicing factors, particularly SRSF1, is hyper-phosphorylated in LigI-deficient cells and this modification is accompanied by a shift in the alternative splicing program of apoptotic genes such as caspase 9 (Leva et al., 2012). Notably, both the level of SRSF1 phosphorylation and splicing programs can revert to those observed in normal cells by inhibiting ATM activity, indicating that SRSF1 phosphorylation could be part of a regulatory circuit through which cells cope with DNA damage. In agreement with this interpretation, SRSF1 phosphorylation is modulated in response to a wide set of DNA-damaging insults (Leva et al., 2012) and SRSF1 is involved in the choice between pro- and anti-apoptotic pathways (Moore et al., 2010).

Phosphorylation of SRSF1 is also relevant to prevention of DNA damage induced by transcription through a process referred to as transcription-associated mutagenesis, or TAM, in which DNA damage preferentially occurs on the non-transcribed strand of DNA. Moreover, transcription can promote transcription-associated recombination (TAR), which is largely, but not exclusively (Wahba et al., 2011), due to transcription-replication conflicts generated by topological constraints. According to the twin-domain model (Liu and Wang, 1987), negative and positive supercoiling domains are transiently generated behind and ahead, respectively, of the moving transcription complex during elongation: positive supercoils can impede further DNA unwinding, whereas excessive negative supercoiling favors the opening of the duplex DNA and facilitates the hybridization of the nascent RNA molecule to the template giving rise to the so-called R-loops. One of the factors that prevents the formation of R-loops is Topo I, which relaxes super-helical stress in duplex DNA. Topo I limits R-loop formation by targeting RNA splicing and RNP assembly factors; particularly SRSF1, which appears to function in the same pathway as Topo I in preventing replication stress (Tuduri et al., 2009). The connection between Topo I and SRSF1 was suggested for the first time in 1996 by Jamal Tazi (Rossi et al., 1996) who identified Topo I as a specific kinase for SRSF1. The kinase activity of Topo I is controlled by poly(ADP-ribose) – PAR – which shifts Topo I from SRSF1 phosphorylation to DNA cleavage. Interestingly, Topo I, SRSF1, and PAR-polymerase display a high affinity for the phosphorylated CTD of RNAPII. It has been proposed that the equilibrium between these factors is relevant both for the capacity of Topo I to relieve the torsional stress generated by RNAPII and to phosphorylate SRSF1 engaged in cotranscriptional splicing events (Malanga et al., 2008). The spacer between the two RRMs of SRSF1 appears to have an important role in this phenomenon since it controls both phosphorylation of the RS domain and DNA nicking activity of Topo I. In fact, the spacer is crucial for the positioning of RRM2 in the cavity normally occupied by DNA (Ishikawa et al., 2012). It has been proposed that this interaction may be modulated by other events that involve the spacer, namely the interaction with the mRNA export factor TAP and the methylation of two arginine residues, a post-translational modification that can also impact the subcellular localization of SRSF1 (Sinha et al., 2010).

Phosphorylation of SR factors is also relevant to modulate the splicing profile of TAF1 in response to DNA damage. TAF1 is a subunit of the general transcription factor TFIID and is required for RNAPII activity. Via alternative splicing the *Drosophila melanogaster* *TAF1* gene produces four mRNAs, TAF1-1 to 4. Interestingly, both IR and CPT promote the expression of TAF1-3 and TAF1-4 isoforms. However, the response to IR is mediated by ATM and CHK2, while the effect of CPT requires ATR and CHK1 (Katzenberger et al., 2006). The mechanism underlying this splicing decision is still unidentified. It has been proposed that AKT, a protein kinase which plays an important role in cell survival is involved. ATM mediates full activation of AKT in response to IR (Viniegra et al., 2005), and in turn AKT regulates the function of SR splicing factors by phosphorylating the RS domain (Blaustein et al., 2005).

Another example involves the regulated phosphorylation and acetylation of the SR protein SRSF2 (also called SC35). Acetylation on Lys52 in the RRM inhibits RNA binding and promotes proteasomal degradation. This modification is controlled by the competing activities of the acetyl transferase TIP60 and the deacetylase HDAC6. DNA-damaging agents such as cisplatin inhibit TIP60 expression and increase SRSF2 stability. TIP60 also controls nuclear translocation of the SR kinases SRPK1 and SRPK2, which induce phosphorylation of SR proteins and control their localization and activity. Thus, cisplatin-induced loss of TIP60 leads to the accumulation of non-acetylated, phosphorylated SRSF2, which in turn promotes the production of the pro-apoptotic splicing isoform of caspase-8 (Edmond et al., 2011). This analysis provides an exciting example of how multiple post-translational modifications and regulated proteasomal degradation of a splicing factor cooperate to promote apoptosis in response to DNA damage. Consistent with its crucial role in the activation of the apoptotic splicing program of genes such as c-flip, caspases-8, -9, and Bcl-x, the expression of SRSF2 increases in response to DNA damage. Interestingly, SRSF1 and SRSF2 appear to have antagonistic activities with SRSF1 favoring anti-apoptotic splicing while SRSF2 promotes apoptosis. Consistent with this interpretation, SRSF2 gene transcription is controlled by E2F1, which promotes apoptosis through both transcription-dependent and -independent mechanisms (Merdzhanova et al., 2008).

In addition to phosphorylation, other post-translational modifications are relevant to activity modulation of RBPs in the DDR. An example comes from the analysis of hnRNP K, a protein crucial for IR-induced cell cycle arrest. HnRNP K cooperates with p53 in transcriptional activation of cell cycle arrest genes such as 14-3-3, GADD45, and p21, in response to DNA damage (Moumen et al., 2005). hnRNP K is a substrate of the ubiquitin E3 ligase MDM2 and, upon DNA damage, is de-ubiquitylated and sumoylated on Lys 422 in the KH3 domain. This modification is regulated by the E3 ligase polycomb Pc2/CBX4 and is required for p53 transcriptional activation. Abrogation of hnRNP K sumoylation leads to aberrant regulation of the p53 target gene p21 (Lee et al., 2012; Pelisch et al., 2012). Many other hnRNPs are SUMO substrates (Vassileva and Matunis, 2004) raising the possibility that this modification is important to modulate the activity of RBPs in response to DNA damage. For instance, sumoylation of hnRNP F could be relevant to the activity of hnRNP H/F in p53 pre-mRNA 3′-end processing, protein expression, and p53-mediated apoptosis (Decorsière et al., 2011).

## RBPs MAY DIRECTLY PARTICIPATE IN THE DDR

A few RBPs have a dual life; they are associated both with complexes involved in RNA metabolism and with the DNA repair machinery. This condition reflects the fact that proteins assemblies involved in transcription, splicing, and DNA repair frequently operate on the same tract of a DNA molecule. To date no one has investigated whether the recruitment of RBPs to the DNA repair complex is evidence that RNA molecules may play a role in genome stability programs as recently suggested by the discovery of short non-coding RNAs complementary to sites of DNA damage (Francia et al., 2012). Below we report a few examples of RBPs interacting with DNA repair assemblies.

### hnRNP G/RBMX

A recent genome-wide screening for regulators of HR (Adamson et al., 2012) identified hnRNP G as a positive regulator that transiently accumulates at sites of DNA damage. This finding raises the possibility (shared with other RBPs such as hnRNP C and hnRNP K) of a direct role in the DDR. The biochemical consequences of transiently accumulating hnRNP G at sites of damage remains to be determined. The authors hypothesized that the recruitment of hnRNP G could help bundle PAR (polyADP-ribose) structures and hold breaks together.

### Ntr1/Spp382

Non-homologous end joining (NHEJ) in mammalian and yeast cells requires a set of common core factors, including the DNA end-binding proteins Ku70 (Ku70p) and Ku80 (Ku80p), as well as the DNA ligase LIG4 (Dnl4p) and its associated factor XRCC4 (Lif1p; Sancar et al., 2004). Recently, it has been shown that both human XRCC4 and its yeast homologue Liflp interact with the putatively orthologous G-patch proteins Ntr1p/Spp382p and NTR1/TFIP11 that have recently been implicated in spliceosome disassembly (Fourmann et al., 2013). G-patches are short conserved sequences of about 40 amino acids containing seven highly conserved glycine residues that have been proposed to mediate RNA binding (Aravind and Koonin, 1999). The interaction with NTR1 (Ntr1p) prevents the formation of an active enzyme complex between XRCC4 (Lif1p) and LIG4 (Dnl4p) thus reducing NHEJ efficiency (Herrmann et al., 2007).

### SFPQ/PSF

SFPQ (splicing factor proline and glutamate-rich), also known as PSF (polypyrimidine tract-binding protein-associated splicing factor) and its paralogs p54nrb/non-POU domain containing octamer binding (NONO) and Paraspeckle Component 1-PSPC1 are members of the *Drosophila* behavior/human splicing (DBHS) family and components of sub-nuclear bodies called paraspeckles (Shav-Tal and Zipori, 2002). SFPQ/PSF has a direct role in the DDR that involves its ability to bind and modulate the function of RAD51 a key component of the HR pathway (Rajesh et al., 2011). Interestingly, SFPQ/PSF has DNA re-annealing and strand-invasion activity that may lead to the formation of D-loop structures similar to intermediates observed during HR (Akhmedov and Lopez, 2000). It has not yet been investigated if this protein can also interact with R-loops, which appear to be deleterious for genome stability.

SFPQ and its highly similar (71% identity) paralog NONO form a heterodimer involved in various aspects of RNA metabolism, such as transcription, pre-mRNA processing, and transcription termination. They are also implicated in nuclear retention of hyper-edited RNA (Passon et al., 2012). In this function they act together with Matrin 3 (MATR3) a highly conserved, inner nuclear matrix and RBP, which is a target of ATM and CHK1 (Blasius et al., 2011). A SFPQ/NONO complex promotes NHEJ *in vitro*, and is probably involved in DSB repair in vertebrates (Bladen et al., 2005). In agreement with this idea, attenuation of NONO expression impairs DSB repair and increases radiation-induced chromosomal aberrations (Li et al., 2009). Moreover, SFPQ/NONO is rapidly recruited to sites of DNA damage induced by laser microbeams and its release from these sites is regulated by MATR3 (Salton et al., 2010).

### PRP19

PRP19/PSO4 is a multifunctional protein also known as nuclear matrix protein 200 NMP200 (Gotzmann et al., 2000), UBOX4 for its involvement in the ubiquitin pathway (Hatakeyama et al., 2001), and senescence evasion factor SNEV (Grillari et al., 2005). The PRP19 complex consists of four polypeptides that form a salt-stable core (CDC5L, PRLG1, Prp19, and SPF27) with three more loosely associated polypeptides (HSP73, CTNNBL1, and AD002; Grote et al., 2010). PRP19 is found at the core of catalytically activated spliceosomes (Grote et al., 2010) and its ubiquitin ligase activity plays a critical role in activation of the spliceosome (Song et al., 2010).

The first indication that PRP19 had a role in the DDR was the identification of the pso4-1 mutant in *S. cerevisiae* that displays increased sensitivity to the DNA cross-linking drug psoralen. This mutant shows defects in some types of recombination, including gene conversion, crossing over, and intrachromosomal recombination. It belongs to the RAD52 epistasis group for strand-break repair and its product participates in the DNA rejoining step of the repair of cross-link lesions (de Morais et al., 1996). In human cells, Prp19 is strongly up-regulated in response to DNA damage and its down-regulation results in DSBs, apoptosis, and reduced survival after exposure to IR. Moreover, Prp19 is a target of post-translational modifications elicited by DNA damage. The human protein is phosphorylated at S149 by ATM in response to oxidative stress and DSB-inducing agents (Dellago et al., 2012). DNA damage also induces ubiquitination of PSO4 and this modification disrupts the interaction with both CDC5L and PLRG1. Interestingly, in further support of its involvement in the DDR, the CDC5L subunit of the complex directly interacts with the master checkpoint kinase ATR (Legerski, 2009).

Recently, PRP19 has been implicated in the transcription-coupled repair (TCR) pathway, which deals with DNA damage that blocks transcription elongation. This activity of PRP19 depends on the interaction with XAB2 [xeroderma pigmentosum group A protein (XPA) binding protein 2], a molecular partner of XPA, that interacts also with Cockayne syndrome group A and B proteins (CSA and CSB) and RNAPII and it is involved both in TCR and transcription (Kuraoka et al., 2008).

Recent studies have started to uncover the intricacy of interactions between complexes once considered completely unrelated. Thus, XAB2 (also known as Syf1) mediates the interaction of PRP19 with RNAPII and is responsible for its role in TCR. In turn, the PRP19 complex is necessary for the recruitment of the THO/TREX complex to transcribed genes, which is important to prevent the formation of R-loops and genome instability. The spliceosome is a highly dynamic molecular machine that is assembled in a stepwise manner onto the pre-mRNA, leading to the formation of intermediates called complexes E, A, B, B*, and C (Wahl et al., 2009). The first trans-esterification generates the C complex, which catalyzes the second step of the splicing reaction. Interestingly, human PRP19 complexes containing XAB2/hSyf1 are present within the B complex, whereas THO/TREX components are only present in the C complex (Chanarat et al., 2011; see **Figure 3B**).

The role of RBPs in DNA repair is still largely unexplored, probably because it has been underestimated by scientists working both in the RNA and DNA repair fields. However, the list of RBPs involved in DNA repair or colocalizing with sites of DNA damage is continuously growing, which clearly points to the existence of relevant connections between the two processes. In the last decade, transcription, RNA and RNP complexes have been implicated in epigenetic processes (Burgess et al., 2012). We are tempted to propose that they may play a similar role in the profound higher-order reorganization associated with DNA repair. As a matter of fact short non-coding RNAs complementary to sequences flanking DSBs have been recently described and shown to control DDR activation at sites of DNA damage (Francia et al., 2012). Generation of these RNAs requires the activity of Drosha and Dicer two ribonucleases involved in the RNAi pathway. However, nothing is known about the nature and synthesis of the precursor RNA molecules. Whether the RBPs listed above may have a role in this process is an open and intriguing possibility.

## RBPs MAY PREVENT DNA DAMAGE

### A ROLE FOR mRNP BIOGENESIS FACTORS IN PREVENTING HAZARDOUS R-LOOPS

**FIGURE 4:**
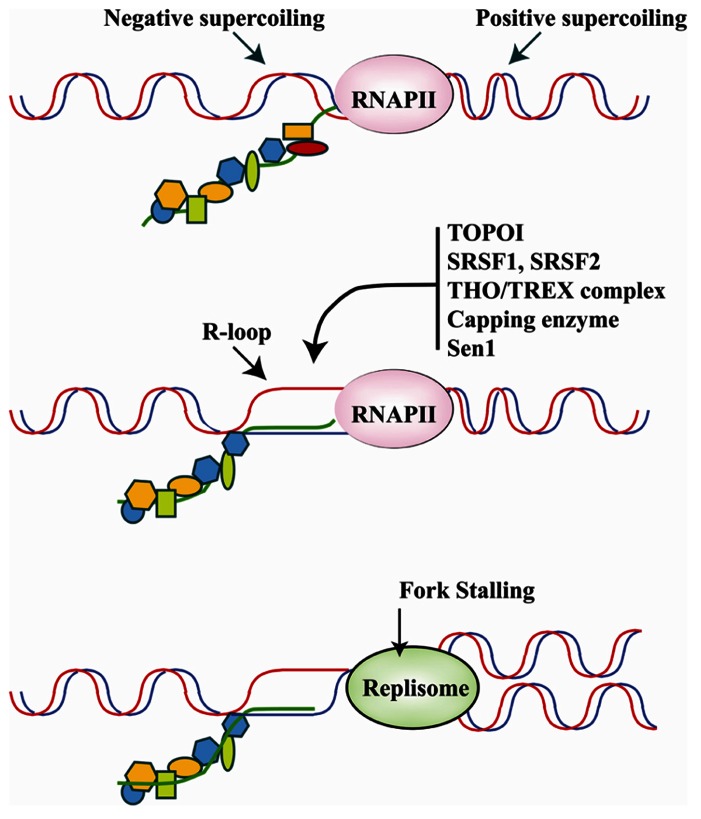
**Upper panel: the translocation of the transcriptional apparatus along the DNA induces positive and negative supercoiling, respectively, in front of and behind RNAPII.** The physiological association of RBPs with the pre-mRNA molecule as it emerges from the transcriptional machinery is believed to play a major role in counteracting R-loop formation in negatively super-coiled regions. Middle panel: hybridization of the nascent RNA with the DNA template results in the formation of R-loops and occurs upon down-regulation, inhibition or mutation of several specific RBPs involved in different steps of pre-mRNA synthesis/maturation. The list of RBPs that may influence R-loop formation includes Capping enzymes, splicing factors SRSF1 and SRSF2, the THO/TREX complex, and Sen1/senataxin which is important for transcription termination. Moreover, DNA topoisomerase I (Topo I) by relieving torsional stress and phosphorylating SRSF1 can prevent R-loop formation. Bottom panel: R-loops hamper the movement of the DNA replication fork, which promotes genome instability. Thus, RBPs may be crucial to genome stability programs by inhibiting R-loop formation.

A transcriptional R loop is a structure in which a nascent transcript is partially or completely hybridized with the template strand leaving the other strand unpaired (Huertas and Aguilera, 2003). The topology of the template DNA (i.e., accumulation of negative supercoiling behind the transcriptional apparatus), and the DNA sequence (i.e., G-richness) significantly influence the formation and size of RNA–DNA hybrids in *in vitro* reactions, suggesting that the capacity to form R-loops is an inherent property of the nascent RNA molecule. R-loops are highly mutagenic structures. The unpaired DNA strand in an R-loop, in fact, is more sensitive to DNA-damaging agents and nucleases and, as in the case of B cell immunoglobulin class switching, is targeted by activation-induced cytidine deaminase (AID)-mediated DNA cytosine deamination (Gómez-González and Aguilera, 2007). Moreover, the R-loop is highly recombinogenic and can generate a block for incoming DNA replication forks or even provide unscheduled RNA primers for DNA polymerases (Bermejo et al., 2012; **Figure 4**).

Because of their negative effects on genome stability, several mechanisms operate to avoid R-loop formation. First of all, as indicated by structural studies, the nascent transcript that emerges from the exit channel of the RNA polymerase has already separated from the template DNA strand. This also implies that R loops do not directly extend from the transcription bubble (Westover et al., 2004). Topoisomerases, mainly Topo I, are active during transcription to prevent the accumulation of negative supercoiling behind the RNAPII that are suitable for R-loop formation. Moreover, RNase H activities operate to reduce the level of RNA:DNA hybrids (Wahba et al., 2011). Finally, the nascent RNA molecule, as soon as it emerges from the transcriptional apparatus is sequestered in RNP complexes. How this protein–RNA packaging influences the capacity to form R-loops is still a matter of investigation even if it is commonly assumed that binding to RBPs is alternative to R-loop formation (Huertas and Aguilera, 2003). Notably, splicing proteins have been selected in screening performed in mammalian cells designed to identify factors able to prevent spontaneous DNA breaks (Paulsen et al., 2009). On the other hand, experimental evidence suggests that R-loops physiologically form within the cells. Indeed, R-loop structures play physiological roles in immunoglobulin class switch recombination (CSR) in human B cells (Yu et al., 2003) and in the promotion of transcription termination, as in the case the human β-actin gene (Skourti-Stathaki et al., 2011). Moreover they may occur during transcription of very long genes whose transcription may take more than one cell cycle (Helmrich et al., 2011).

RNA polymerase II plays a critical role in the processing of mRNA precursors (pre-mRNA). This involves the interaction of a large number of factors involved in capping, splicing and termination/poly-adenylation. The interaction is mediated by the CTD of RNAPII, which is composed mainly of a repeated heptapeptide motif, YSPTSPS, that is extensively phosphorylated during the transcription cycle (Schroeder et al., 2000). Phosphorylation of serine-5 in the heptad repeats by human Cdk7 and yeast Kin28, occurs shortly after transcription initiation and is required for cotranscriptional recruitment of pre-mRNA processing factors. Several studies have indicated that all these factors may have a role in preventing the formation of R-loops.

Interestingly, under certain circumstances some of these factors may actually promote R-loop formation. This is the case of Capping Enzymes whose interaction with the transcription machinery is critical for RNA elongation. *In vitro*, in the presence of phosphorylated CTD, the human Capping enzymes specifically promote formation of R-loops. There is no evidence so far that Capping enzymes are involved in DNA damage. Intriguingly, however, their *in vitro* capacity to induce R-loops is antagonized by splicing factor SRSF1 (Kaneko et al., 2007), whose down-regulation *in vivo* promotes R-loop formation leading to DNA fragmentation and cell death (Li et al., 2005).

### THO/TREX

One of the best-characterized RBP complexes shown to prevent R-loop formation is called THO/TREX. The multimeric THO/TREX complex is conserved throughout evolution and human homologues of all the yeast components have been identified: hTHO2/THOC2, hHpr1/THOC1, fSAP79/THOC5, fSAP35/THOC6, fSAP24/THOC7, and hTex1/THOC3, the DEAD-box RNA helicase Sub2/UAP56 and the mRNA export adaptor protein Yra1/Aly/THOC4 (Masuda et al., 2005). Both in yeast and humans the complex is functionally involved in connecting transcription, mRNP biogenesis and genome instability. Its mutation increases R-loop-dependent genome instability, and in the mouse enhances class-switching recombination in the immunoglobulin locus (Domínguez-Sánchez et al., 2011). The analysis of mutated THO/TREX in yeast by Aguilera and colleagues (Huertas and Aguilera, 2003) provided the first evidence that RNA metabolic functions have a role in preventing R-loops and that these structures mediate both impairment of transcription elongation and TAR. Using an engineered transcript containing a hammerhead ribozyme, they showed that the nascent mRNA itself has a role in the origin of transcription elongation impairment and genome instability associated with THO mutations. The function of THO/TREX-2 complexes is to couple RNAPII transcription (Gómez-González et al., 2011) with mRNA export through the nuclear envelope, a process known as gene gating. In this way another topological constraint is superimposed on to DNA during the transcription process. The ATR checkpoint phosphorylates key nucleoporins to counteract gene gating, thus neutralizing the topological tension generated when forks encounter gated genes (Bermejo et al., 2011). Mutants in some elements of this pathway may eventually lead to formation of R-loops and DNA damage. Thus, one physiological function of factors such as the THO/TREX-2 complexes would be to prevent R-loop formation and relieve topological constraints (Bermejo et al., 2012).

### THSC COMPLEX

THSC (Thp1-Sac3-Sus1-Cdc31) is another complex that, similar to THO/TREX, connects transcription elongation to mRNA export via a RNA-dependent dynamic process. The THSC complex is formed by Thp1, Sac3, and Sus1 Cdc13 subunits and was previously shown to interact with the SAGA (Spt-Ada-Gcn5-Acetyltransferase) complex, a histone acetyltransferase. However, its role in transcription elongation is independent of SAGA and is linked to mRNA export. It has been proposed that a feedback mechanism exists by which improperly formed mRNPs, presumably stacked at the nuclear pore, have a backward effect promoting transcription impairment and genetic instability (González-Aguilera et al., 2008).

### SPLICING FACTORS OF THE SR FAMILY

In addition to the THO/TREX complex, several mRNP biogenesis/export factors, from yeast to humans, cause TAR when mutated or down-regulated, even though their effect is weaker than that observed with THO/TREX mutants (Luna et al., 2005). Genetic studies in yeast proved that deletions of genes acting at various stages of RNA metabolism, from transcription initiation to RNA degradation and export, increase the rate of instability 4- to 16-fold over wild type (Wahba et al., 2011). In mammals, Manley’s group has clearly proved that a specific subset of splicing factors of the SR family, including SRSF1 (Li et al., 2005) SRSF2 and SRSF3, can inhibit the formation of R-loops (Li and Manley, 2005). Thus, errors in RNA processing pose a major threat to genome integrity. In human and chicken DT40 cells, SRSF1 prevents R-loop formation (Li et al., 2005). A screen for suppressor(s) of SRSF1 depletion-induced genome instability in chicken DT40 cells identified RNPS1, a nuclear RBP with multiple roles in mRNA maturation. The fact that RNPS1 cannot compensate for SRSF1 function in splicing, suggests that the ability to prevent R-loops is a distinctive feature of only a few RBPs, which is separate from their activity in splicing (Li et al., 2007).

It is commonly accepted that R-loops are prevented by specific RBPs that facilitate the proper packaging of nascent mRNA into RNP particles, which in turn would strongly reduce the ability of the RNA molecule to rehybridize with the transiently opened DNA strands behind the RNAPII (Huertas and Aguilera, 2003). However, it is possible that dysfunction of mRNA processing factors may enhance R-loop formation by increasing RNA half-life, by blocking transcription elongation and possibly stabilizing negative supercoiling, or by impairing 3′-end processing and/or termination that would affect RNA release from the transcription site. Another major point of discussion concerns the mechanism through which R-loops favor genome instability. However, the majority of scientists favor the idea that R-loop-mediated genomic instability is mainly caused by impairment of replication fork progression (Tuduri et al., 2009; Gan et al., 2011). Strong support of this hypothesis comes form the analysis of Sen1/Senataxin.

### Sen1/SENATAXIN

Recent data suggest that R-loops may play a physiological function in transcription termination. Two classes of terminator sequences have been identified in human genes: cotranscriptionally cleaved (CoTC) RNA sequences and transcription pause sites. The latter correspond to G-rich sequence elements and act to slow down elongating RNAPII. They have been identified in several human genes including the human β-actin gene (Skourti-Stathaki et al., 2011). Sen1 is a conserved RNA/DNA helicase known to cooperate with Xrn2/Rat1 in promoting efficient transcriptional termination in *S. cerevisiae* (Kawauchi et al., 2008). This function is conserved in evolution. Indeed, depletion of human senataxin, the mammalian Sen1 homolog, increases RNAPII density downstream of the poly(A) site and induces R-loop formation. Taking into account the behavior of the Sen1 mutant in yeast (Mischo et al., 2011) and the effect of senataxin inactivation in humans cells at the β-actin gene locus (Skourti-Stathaki et al., 2011), it has been suggested that *in vivo* R-loops may be more common than previously believed and their unwinding by Sen1/senataxin is physiologically important for transcription termination at transcriptional pause sites. Recent data indicate that pathological R-loops in Sen1 mutants would induce hyper-recombination *via* inhibition of DNA replication. From this viewpoint Sen1 would be relevant to protect genome integrity from DNA damage resulting from the head-on collision of transcription and replication. This property seems to be linked to the association of Sen1 with DNA replication forks which is crucial to protect fork integrity across RNAPII-transcribed genes (Alzu et al., 2012). A similar role in preventing replication-transcription conflicts was proposed for human senataxin (Yüce and West, 2013). Finally, as a further example of the connections between complexes involved in pre-mRNA synthesis, RNA processing, DNA repair and replication, Sen1 has been involved also in TCR via an interaction with Rad2, the yeast homologue of human XPG (xeroderma pigmentosum complementation group G; Ursic et al., 2004).

## PERSPECTIVES

DNA damage induces the activation of signaling pathways that target the expression and post-translational modification of RBPs involved in the metabolism of protein-coding transcripts. However, the physiological consequences of these events are far from being understood even though it is highly probable that targeting of RBPs may impact gene expression profiles. For instance, solid evidence bolsters the idea that the recruitment of specific splicing factors, such as SRSF1 and SRSF2, in the DDR can be linked to the inhibition/activation of apoptotic pathways. An appealing hypothesis is that this strategy could have a broader effect on gene expression and cell differentiation programs.

A growing body of data from the last ten years implicates the DDR in regulating precursor or stem cell differentiation programs (Sherman et al., 2011). One clear example is the development of vertebrate adaptive immune systems that requires the programmed induction and subsequent repair of DSBs during antigen receptor gene rearrangements to assemble a complete Ig gene *via* V(D)J recombination. The response to these programmed DSBs elicits ATM-dependent and ATM-independent mechanisms that ultimately control the expression of approximately 300 genes, a significant fraction of which regulates cellular processes important for lymphocyte development (Bredemeyer et al., 2008). Notably, several of these genes are regulated in response to genotoxic DNA damage as well, indicating that unphysiological DSBs disrupt normal cellular functions by altering specific gene expression programs (Bredemeyer et al., 2008). DSBs are also necessary for CSR and somatic hypermutation (SHM) required for the production of high-affinity antibodies of different isotypes. During CSR, production of DSBs requires the programmed formation of R-loops (Roy et al., 2008) and deoxycytidine deamination mediated by AID (Chaudhuri et al., 2007). The response to DSBs produced by AID activates an ATM-dependent signaling pathway that regulates a network of genes involved in proliferation, B-cell self-renewal, and cell differentiation (Sherman et al., 2010). Interestingly, unscheduled AID-mediated DSBs are implicated in cancer (Park, 2012) even though it is unclear if the link with cancer involves targeting of aberrant R-loop structures.

DNA damage response may also influence differentiation of pluripotent embryonic stem cells (ESCs). DNA lesions in ESCs could be particularly harmful for the organisms. Thus, apoptotic pathways may clear severely damaged cells from the replicating stem cell pools. Alternatively, ESCs can activate a gene expression program controlled by p53 to promote cell cycle withdrawal and differentiation (Hong et al., 2009). Two parameters appear to be crucial to determine the choice between apoptotic vs. differentiation programs. The first one is related to the extent of DNA damage in the sense that apoptosis or senescence is the proper response to extensive genome-wide damage. A low level of DNA damage may induce cell differentiation programs as in developing B cells during Ig gene modifications. A second decision-regulating process is linked to the differentiation state of cells that experience DNA damage. For example, DDR signaling *via* ATM promotes the quiescence of stem cells, whereas in more advanced lineage progenitors, such as pre-B (Bredemeyer et al., 2008) cells, ATM-dependent DDR signaling promotes cell differentiation.

We speculate that, because of their involvement in gene expression programs, RBPs may have a role in all these decisions. Moreover, in view of their association with large, still poorly characterized, multiprotein assemblies that link genome stability, transcription, and pre-mRNA processing, RBPs may be crucial not only for the response to genotoxic stress but also for the programmed induction of DNA damage during cell differentiation processes.

## Conflict of Interest Statement

The authors declare that the research was conducted in the absence of any commercial or financial relationships that could be construed as a potential conflict of interest.
